# Self-audits as alternatives to travel-audits for improving data quality in the Caribbean, Central and South America network for HIV epidemiology

**DOI:** 10.1017/cts.2019.442

**Published:** 2019-12-26

**Authors:** Sarah C. Lotspeich, Mark J. Giganti, Marcelle Maia, Renalice Vieira, Daisy Maria Machado, Regina Célia Succi, Sayonara Ribeiro, Mario Sergio Pereira, Maria Fernanda Rodriguez, Gaetane Julmiste, Marco Tulio Luque, Yanink Caro-Vega, Fernando Mejia, Bryan E. Shepherd, Catherine C. McGowan, Stephany N. Duda

**Affiliations:** 1 Department of Biostatistics, Vanderbilt University School of Medicine, Nashville, TN, USA; 2 Departamento de Pediatria, Universidade Federal de Minas Gerais, Belo Horizonte, Brazil; 3 Departamento de Pediatria, Universidade Federal de São Paulo, São Paulo, Brazil; 4 Instituto Nacional de Infectologia Evandro Chagas, Rio de Janeiro, Brazil; 5 Unidad Médica, Fundación Arriarán, Santiago, Chile; 6 Le Groupe Haïtien d’Etude du Sarcome de Kaposi et des Infections Opportunistes, Port-au-Prince, Haiti; 7 Instituto Hondureño de Seguridad Social and Hospital Escuela Universitario, Tegucigalpa, Honduras; 8 Departamento de Enfermedades Infecciosas, El Instituto Nacional de Ciencias Médicas y Nutrición Salvador Zubirán, Mexico City, Mexico; 9 Instituto de Medicina Tropical Alexander von Humboldt, Universidad Peruana Cayetano Heredia, Lima, Peru; 10 Division of Infectious Diseases, Department of Medicine, Vanderbilt University School of Medicine, Nashville, TN, USA; 11 Department of Biomedical Informatics, Vanderbilt University School of Medicine, Nashville, TN, USA

**Keywords:** Observational data, data quality, source document verification, validation, data audits

## Abstract

**Introduction:**

Audits play a critical role in maintaining the integrity of observational cohort data. While previous work has validated the audit process, sending trained auditors to sites (“travel-audits”) can be costly. We investigate the efficacy of training sites to conduct “self-audits.”

**Methods:**

In 2017, eight research groups in the Caribbean, Central, and South America network for HIV Epidemiology each audited a subset of their patient records randomly selected by the data coordinating center at Vanderbilt. Designated investigators at each site compared abstracted research data to the original clinical source documents and captured audit findings electronically. Additionally, two Vanderbilt investigators performed on-site travel-audits at three randomly selected sites (one adult and two pediatric) in late summer 2017.

**Results:**

Self- and travel-auditors, respectively, reported that 93% and 92% of 8919 data entries, captured across 28 unique clinical variables on 65 patients, were entered correctly. Across all entries, 8409 (94%) received the same assessment from self- and travel-auditors (7988 correct and 421 incorrect). Of 421 entries mutually assessed as “incorrect,” 304 (82%) were corrected by both self- and travel-auditors and 250 of these (72%) received the same corrections. Reason for changing antiretroviral therapy (ART) regimen, ART end date, viral load value, CD4%, and HIV diagnosis date had the most mismatched corrections.

**Conclusions:**

With similar overall error rates, findings suggest that data audits conducted by trained local investigators could provide an alternative to on-site audits by external auditors to ensure continued data quality. However, discrepancies observed between corrections illustrate challenges in determining correct values even with audits.

## Introduction

High-quality data are essential for valid inference and decision-making in observational HIV cohort research. Source document verification, or data auditing, is the standard for ensuring high-quality data in clinical trials [1] and has also been used to assess data quality in observational studies [2–7]. The data audit process involves a group of external data auditors visiting the research site, comparing records in the research database to clinical source documents and reporting any discrepancies. In addition to assessing the accuracy and completeness of existing data, audits can help educate local staff on good data management practices, highlight areas for improvement in data collection methods and provide a deterrent against data fraud. Statistical methods have been developed that incorporate audit information into analyses, potentially providing more accurate estimates based on error-prone data [8].

Despite its benefits, source document verification of the entire database, or even of a subset of records and variables, is a resource-intensive exercise that often exceeds the available capacity and budget of research studies. We have developed methodologies and tools to simplify the audit preparation and feedback process [6,9], but the practice remains relatively uncommon among observational HIV cohorts. Although the most objective audits are conducted by external auditors, sending trained auditors to distant sites within multinational networks (on-site monitoring or travel-audits) requires extensive travel funds and dedicated personnel effort [10]. External auditors require additional time to familiarize themselves with local source documents and procedures, while sites may spend unplanned time obtaining patient charts for review and hosting the visitors. Language differences can further complicate the audit process.

To address the logistical and financial challenges of these travel-audits, the present work investigates the efficacy of audits executed by local sites themselves, referred to as “self-audits.” These self-audits explore a creative way to continue maintaining high data quality standards, while markedly lowering the costs of performing the audits. Several novel internal checks are built into self-audits in an attempt to strengthen their validity: (1) records to be audited are randomly selected by the data coordinating center rather than the sites themselves, and (2) prior to performing their self-audits, local personnel are notified that several sites will be randomly selected to have their self-audits verified by travel-auditors, that is, that external auditors will travel to some of the sites and validate the same records as self-auditors. We describe our experience conducting a self-audit process within a multinational HIV cohort and compare the findings from self- and travel-audits at those sites randomly selected to receive both.

## Materials and Methods

### Cohort

The Caribbean, Central, and South America network for HIV epidemiology (CCASAnet) is a consortium of HIV care and treatment clinics in seven countries in Latin America. CCASAnet clinics pool their routine clinical care data to conduct collaborative research to better understand the HIV epidemic in the region [11]. In early 2017, investigators from eight sites (six adult and two pediatric) participated in a new self-audit process to review their data. Participating sites included Instituto Nacional de Infectologia Evandro Chagas in Rio de Janeiro, Brazil; Universidade Federal de Sao Paulo, Brazil; Universidade Federal de Minas Gerais, Brazil; Fundación Arriarán in Santiago, Chile; Le Groupe Haïtien d’Etude du Sarcome de Kaposi et des Infections Opportunistes in Port-au-Prince, Haiti; Instituto Hondureño de Seguridad Social and Hospital Escuela Universitario in Tegucigalpa, Honduras; El Instituto Nacional de Ciencias Médicas y Nutrición Salvador Zubirán in Mexico City, Mexico; and Instituto de Medicina Tropical Alexander von Humboldt in Lima, Peru.

The CCASAnet data structure roughly follows data exchange protocols outlined by the HIV Cohorts Data Exchange Protocol and the International epidemiology Databases to Evaluate AIDS (IeDEA) [12,13]. Variables captured in the following patient forms were audited: basic demographic information typically captured at enrollment (tblBAS), information on visits and vital status (tblVIS and tblLTFU), CD4+ cell count measurements (tblLAB_CD4), HIV viral load measurements (tblLAB_RNA), antiretroviral therapy regimens and dates (tblART), and clinical endpoints (tblCEP). An abbreviated data dictionary for these tables is provided in the Supplementary material (Table S1). Of 28 audited variables, 13 were captured at each patient’s first appointment only (e.g., sex, birthdate), and 15 were collected multiple times per patient during subsequent clinic visits (e.g., weight, height, CD4+ cell count). For clarity, we refer to individual occurrences or measurements of these data values as “data entries.” Clinical source documents were in the form of parallel paper-based forms at each of the study sites.

### Study Design

For each site, the CCASAnet Data Coordinating Center at Vanderbilt (CDCC-VU) selected 40 patient records to be audited. Of these, 20 records were randomly selected among patients enrolling within the previous year to assess the quality of recent data capture, and 20 records were randomly selected among all patients. For the six adult sites, an additional 10 records were chosen among those audited in a previous study [7]. Institutional review board approval was obtained from each site and the CDCC-VU.

Prior to the audit, each site selected representatives to attend a two-hour online training session that explained procedures for conducting a data audit and documenting findings. Following completion of the training session, sites were given two weeks to complete the self-audit. Upon return of self-audit results, sites were compensated $2000 US dollars for their efforts. In June 2017, two CDCC-VU investigators performed on-site audits at three randomly selected HIV clinics (one adult and two pediatric) using published audit procedures [6,9] to audit the same patient records that were chosen for the self-audit. We refer to this as the travel-audit. Travel-auditors spent two and a half days on average performing audits at each site. The self- and travel-audits for this study were completed between May and July 2017.

The CDCC-VU developed a research electronic data capture (REDCap) web-based data capture interface, so site and travel-auditors could view audit records and enter audit findings and data corrections in a structured, electronic format [14]. These REDCap forms were based on audit templates developed in prior audit work [6,9]. The most recently submitted study data for the randomly selected audit patients were imported into the corresponding fields in the REDCap database and displayed during the audit process. Each site could only see its own audit records.

For each data point, both sets of auditors compared the value in REDCap, representing data the site had submitted to CCASAnet for research studies, to the site’s source documents, including paper patient charts, laboratory summaries, and electronic data systems. Within the REDCap interface, auditors were asked to categorize their findings as one of the following: “Value matches the chart (correct),” “Value doesn’t match the chart,” and “Can’t find this value in the chart.” For data entries in error, auditors were prompted to provide corrected values. If they identified a new data entry in the source documents that was not in the REDCap data but should have been, they entered it into a blank supplemental data field with the label “Found value in the chart (was not in the study data).” For our analysis, findings were collapsed into “correct” (matches the chart) and “incorrect” (does not match the chart, could not be found in the chart, or was found in the chart but not present in the study data). Following completion of the audits, audit findings and corrections were extracted from REDCap for analysis.

### Analysis

R Statistical Software (http://www.R-project.org) was used for all analyses, and code is available at http://biostat.mc.vanderbilt.edu/ArchivedAnalyses. Analyses focused on data entries that were reviewed by both sets of auditors, referred to as the “doubly-audited sample.” All patient records were reviewed by self-auditors but due to time constraints many records were not reviewed by travel-auditors. Characteristics of doubly-audited (included) and self-audited only (excluded) patient records were compared using logistic regression models, controlling for site. Because our sample included both pediatric and adult sites, we excluded two variables, receiving prevention of mother-to-child transmission as an infant and birth mode, which are specific to pediatric patients. Entries for clinical AIDS diagnosis prior to first visit, date of prior clinical AIDS diagnosis, and WHO stage were also excluded because they were incompletely audited. (The related variables, prior AIDS diagnosis and date, were included.) All other variables reviewed during self- or travel-audits were included in analyses.

To directly compare findings, we defined an entry in “discordance” to mean that self- and travel-audit findings did not agree. Rates of audit discordance were calculated by variable and by site. Variables collected only once (e.g., sex, birthdate) were calculated as the proportion of patients’ entries in discordance. Repeatedly collected entries (e.g., visit date, height, and labs) were calculated as both the average per-person proportion of entries in discordance and the total percentage of entries in discordance. The Kappa statistic was computed to estimate agreement between all self- and travel-audit entries.

Further descriptive analyses addressed differences in how self- and travel-auditors recorded fixes for incorrect entries, focusing on those agreed to be incorrect by both sets of auditors and excluding those who could not be found in the patient chart. With these, we inspected different fixes submitted for the same incorrect entries (called “mismatched corrections”). Mismatched corrections were reviewed by two investigators to identify possible causes for the mismatch, categorized as audit protocol issues, date approximations, genuine differences, near-equivalent coding, or typographical errors (typos). Audit protocol issues included entries that one team declared incorrect while the other team labeled “could not verify/no source document” (a matter of interpretation or thoroughness of chart searching) or some incomplete data entries that self-auditors corrected in a way that created duplicate data, while travel-auditors labeled them “could not find in the chart” in order to avoid duplicate data rows. For example, an instance of ce_d was corrected by self-auditors to the same date as the previous ce_d (creating a duplicate of this clinical endpoint), while travel-auditors reported that they could not find the original ce_d value. Other correction mismatches occurred because of the combination of dates and date approximation variables. One audit team might record a corrected date “exact to the day,” whereas another recorded a correction that was only “exact to the month.” Although one correction was more precise than the other, both were technically correct.

## Results

**Fig. 1.**
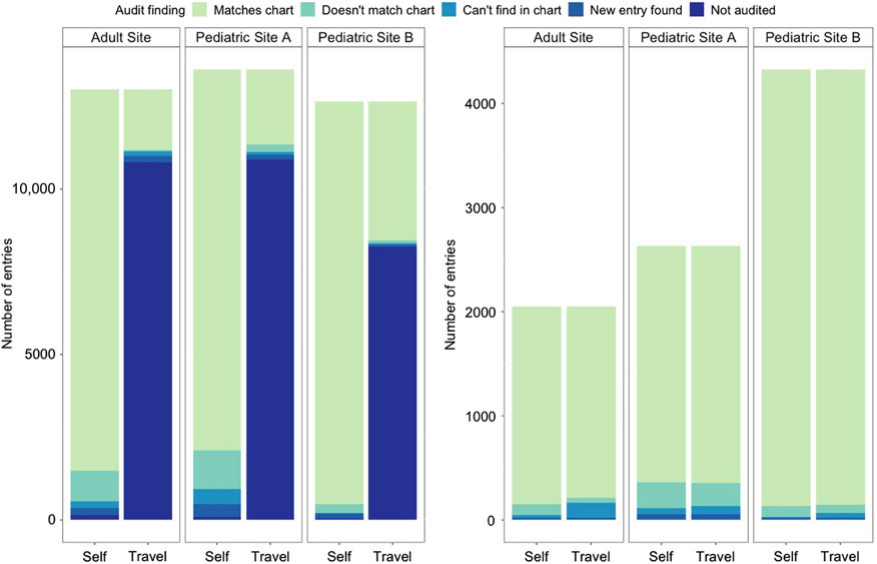
Comparison of audit findings between self- and travel-auditors at the three sites (left) and among only doubly audited entries (right).

A total of 39,269 entries in 130 patient records were selected for self- and travel-audit across the three sites. Figure 1 (left panel) summarizes audit results for these entries. Travel-auditors faced time constraints and therefore audited fewer records; 29,965 of the selected entries (76%) were self- but not travel-audited. Patients whose records were not travel-audited (*n* = 65, 50%) were not materially different from those who were at least partially travel-audited, as the order of auditing was essentially random (Supplementary material Table S2). Among patients who were both self- and travel-audited (*n* = 65), some were not fully audited: there were 52 patients (80%) whose original records contained entries that were self- but not travel-audited and 41 patients (63%) with fields that were travel- but not self-audited. While these percentages appear somewhat similar, only 298 entries (less than 1% of the original sample) were travel- but not self-audited, whereas 29,965 entries (76%) were self- but not travel-audited.

### Overall Data Quality

Across 65 patient records, 8919 data entries capturing 28 clinical and demographic variables were both self- and travel-audited. Figure 1 (right panel) shows the number and distribution of errors by audit site for entries that were both self- and travel-audited. Across all variables, records, and sites, self- and travel-auditors reported similar proportions to be correct (93% vs. 92%) in the doubly audited sample. Self-auditors reported slightly more values not matching the charts (5.0% vs. 3.8%), while travel-auditors assessed that more values could not be found in charts (3.0% vs. 1.0%). Auditors reported the same number of values that were found in the patient chart, but not originally in the database (1.1%).

**Fig. 2.**
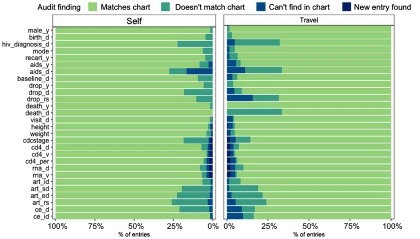
Percentage of audit findings by variable and audit type. Variable definitions are in the Supplemental material.

Figure 2 shows the audit error rates for each variable for both travel- and self-audits among doubly audited patient records. Independently, self- and travel-auditors reported similar rates of incorrect values for all variables in the antiretroviral therapy (ART), viral load, and CD4 data tables (all rates were less than 5% different). From the baseline and visit tables, both teams reported small error rates for sex, death (yes/no), height, weight, and visit date (all less than 5%). However, audit results for date of death substantially differed, as self-auditors reported 0% (0 of 12) incorrect but travel-auditors reported 33% (4 of 12) incorrect. Similar rates of incorrect entries for clinical diagnosis dates (ce_d) were reported by both audit teams, but travel-auditors reported that 16% (30 of 185) of disease codes (ce_id) were incorrect whereas self-auditors reported only 2% (3 of 185) incorrect. Across all variables except AIDS diagnosis date (aids_d), travel-auditors reported a larger number of entries that could not be found in the chart. Details are shown in Table 1.

**Table 1.** Self- and travel-audit discordance by variable in the doubly audited sample (*n* = 8919 entries)

	Audited patient records	Audited entries	Discordant entries	Average discordance per record	Overall discordance
tblBAS^a^	**434**	**434**	**38**	**9%**	**9%**
male_y	65	65	0	0%	0%
birth_d	65	65	1	2%	2%
hiv_diagnosis_d	62	62	14	23%	23%
mode	63	63	5	8%	8%
recart_y	62	62	3	5%	5%
aids_y	36	36	4	11%	11%
aids_d	18	18	5	28%	28%
baseline_d	63	63	6	10%	10%
tblLTFU^a^	**168**	**168**	**18**	**11%**	**11%**
drop_y	52	52	1	2%	2%
drop_d	22	22	4	18%	18%
drop_rs	19	19	8	42%	42%
death_y	63	63	1	2%	2%
death_d	12	12	4	33%	33%
tblVIS^b^	**213**	**4248**	**206**	**11%**	**5%**
visit_d	57	1216	43	6%	4%
height	57	1075	36	5%	3%
weight	57	1061	52	12%	5%
cdcstage	42	896	75	26%	8%
tblLAB_CD4^b^	**164**	**1933**	**46**	**4%**	**2%**
cd4_d	55	652	19	6%	3%
cd4_v	55	649	8	2%	1%
cd4_per	54	632	19	3%	3%
tblLAB_RNA^b^	**96**	**1248**	**54**	**6%**	**4%**
rna_d	48	624	23	8%	4%
rna_v	48	624	31	5%	5%
tblART^b^	**193**	**514**	**69**	**13%**	**13%**
art_id	58	155	9	4%	6%
art_sd	58	153	25	20%	16%
art_ed	39	110	19	17%	17%
art_rs	38	96	16	15%	17%
tblCEP^b^	**100**	**374**	**79**	**20%**	**21%**
ce_d	50	189	48	23%	25%
ce_id	50	185	31	18%	17%

a Variables from tblBAS and tblLTFU are collected once per record.

b Variables from all other tables are repeatedly collected.

For quantitative variables height, weight, lab values, and dates, the median discrepancies between the original entries and the self- or travel-audit corrections (with interquartile range [IQR]) are included in Table 2. Lab dates (cd4_d and rna_d) were corrected in at least 20 entries by either set of auditors, with median corrections of 16 and 17 days from self- and travel-auditors, respectively. For ART regimens, self-auditors submitted corrections for start and end dates of about 1 month on average, while travel-auditors indicated slightly larger fixes to start dates than to end dates (median differences of 28 and 17 days, respectively). Many of the remaining variables were corrected on only a few entries (e.g., birth_d, aids_d, baseline_d, cd4_v, cd4_per), so median discrepancies may appear more extreme. Corrections made to height and weight were minor.

**Table 2.** Magnitude of discrepancies between original entries in quantitative variables found to not match the charts and corrections submitted by self- or travel-auditors

	Self-audit		Travel-audit	
	Corrected entries	Median difference (IQR)	Corrected entries	Median difference (IQR)
tblBAS				
birth_d	3	6 (4, 34) days	2	34 (20, 47) days
hiv_diagnosis_d	14	6 (−377, 110) days	17	14 (−14, 155) days
aids_d	2	116 (43, 190) days	4	10 (−4, 81) days
baseline_d	6	−8 (−17, 2) days	2	817 (416, 1217) days
tblLTFU				
drop_d	3	0 (0, 33) days	1	−309 (NA,NA) days
death_d	0	NA (NA,NA) days	4	7 (−8, 19) days
tblVIS				
visit_d	17	−6 (−31, 29) days	7	−10 (−228, -5) days
height	12	−0.4 (−1.4, 2.9) cm	13	−0.5 (−1.0, 0.5) cm
weight	26	−0.1 (−0.1, −0.1) kg	27	−0.1 (−0.1, −0.1) kg
tblLAB_CD4				
cd4_d	26	17 (1, 38) days	20	16 (1, 40) days
cd4_v	4	−53 (−325, 441)	5	−100 (−600, −5)
cd4_per	8	−0.3 (−15.1, 4.4)%	5	−0.6 (−4.0, 1.0)%
tblLAB_RNA				
rna_d	26	2 (−4, 26) days	31	0 (−8, 27) days
rna_v	18	350 (30, 350)	7	−101 (−498, 366)
tblART				
art_sd	28	32 (0, 154) days	25	28 (0, 47) days
art_ed	23	−26 (−88, 149) days	19	17 (−77, 154) days
tblCEP				
ce_d	36	56 (−2, 147) days	11	30 (21, 227) days

Entries from the doubly audited sample (*n* = 8919) that received audit findings of “doesn’t match chart” from self-auditors and/or from travel-auditors are included in the left and right halves of the table, respectively. IQR, interquartile range; NA, not applicable.

### Comparing Audit Findings

Across all patient entries, 8409 (94%, Kappa = 0.59) received the same assessment from self- and travel-auditors (7988 correct and 421 incorrect). Of the 510 discordant entries, 44% were marked correct by travel-auditors but incorrect by self-auditors, while 56% were marked correct by self-auditors but incorrect by travel-auditors. Patient sex was the only variable with no discordance, but other variables pertaining to CD4 and RNA labs, routine visit variables (visit_d, height, and weight), and baseline variables (recart_y, drop_y, death_y, and birth_d) exhibited less than 5% discordance based on more than 50 entries each.

Variables drop_rs (42%) and death_d (33%) from the follow-up table had the largest individual proportions discordant, but these estimates were based on only 19 and 12 entries, respectively, while many of the more concordant variables were based on >600 entries. Of the eight discordant drop_rs entries, six were originally entered as “Other,” which self-auditors considered matching the chart while travel-auditors corrected three to “LTFU/not known to be dead” and could not find three. Disagreement in the four death_d entries came from self-auditors finding them “correct,” while travel-auditors proposed corrections that were 0, 30, 34, and 14 days from the original entries. There was also 18% discordance in ce_id, with more than half of the discordant ce_id entries unable to be found by travel-auditors but assessed to be correct by self-auditors. Between-audit discordance rates are reported for all variables in Table 1.

Discordance rates for certain variables (e.g., Centers for Disease Control and Prevention [CDC] stage, reason for dropout, dropout date, and death date) differed between the three sites; discordance rates for all variables per site are given in Fig. S1 (Supplemental material). While these variables saw more variability in site-specific discordance rates, we note that rates for reason for dropout, dropout date, and death date were based on a small number of audited entries (no more than 10). We attribute the disparities between discordance rates for CDC stage to differences in the number of audited entries at the three sites: 32 at the adult site and 292 and 572 at pediatric sites A and B, respectively.

### Comparing Audit Fixes

**Fig. 3.**
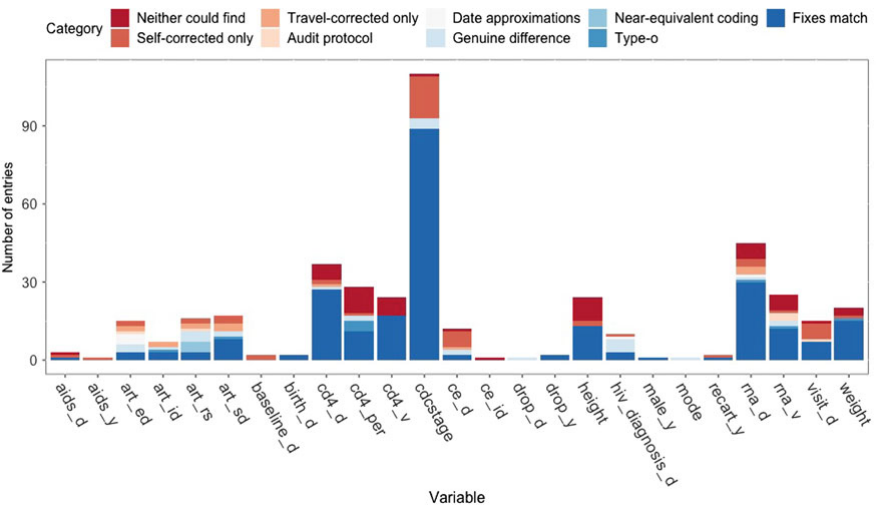
A comparison of corrections made to 421 entries assessed as incorrect by both self- and travel-auditors. This plot includes entries that neither set of auditors could find (which were appropriately left uncorrected), as well as singly and doubly corrected entries.

Of 421 entries marked incorrect by both teams of auditors, 52 (12%) were not corrected by either because the original values could not be found in the patient charts. These are not included when comparing audit fixes, because they were appropriately left uncorrected. Of the remaining 369 erroneous entries, 304 (82%) were corrected by both auditors (called the doubly corrected sample). Most of the singly corrected entries (*n* = 60, 92%) were unable to be found by one team but were found by the other, which resulted in the blank corrections. All entries agreed to be incorrect are included in Fig. 3, colored by the categorized comparison of the self- and travel-auditor corrections.

Of 304 doubly corrected records, 250 (82%) received the same corrections from self- and travel-auditors. While proportions of entries receiving mismatched corrections varied by variable, there were seven variables that received all matching fixes from self- and travel-auditors: CD4 value (*n* = 17), height (*n* = 13), birthdate (*n* = 2), drop from cohort (*n* = 2), prior ART (*n* = 1), date of prior AIDS diagnosis (*n* = 1), and sex (*n* = 1). The mismatched corrections in this sample (*n* = 54) occurred across 15 variables, with the largest number in “reason for changing ART regimen” (*n* = 9), end date of ART regimen (*n* = 8), viral load value (*n* = 6), CD4% (*n* = 6), and date of HIV diagnosis (*n* = 6).

The largest number of mismatches were identified to be genuine differences between self- and travel-corrections (*n* = 29, 54%). These mismatches were found primarily across CDC stage, reason for switching ART regimen, and date of HIV diagnosis variables (4–5 entries each). After this, there were fewer than 10 entries found to be attributable to typographical errors (*n* = 9), differing interpretations of audit protocol (*n* = 6), date approximations (*n* = 6), or near-equivalent coding (*n* = 4). Most typographical errors were found in lab values, where self-auditors entered commas as decimal separators (frequently used throughout South America) instead of periods as decimal point separators (e.g., “11,99” instead of 11.99, with software saving as “1199” when alerts were overridden). Near-equivalent coding applied mostly to the “reason for switching ART regimen” variable (art_rs), where selection of codes depended on auditor interpretation (e.g., “availability of more effective treatment” vs. “simplified treatment available”).

## Discussion

In this study, we describe a novel approach where sites perform self-audits supervised by a central data coordinating center. We compared audit findings between self- and travel-auditors on a sample of 8919 entries across 65 patients capturing 28 HIV variables at three HIV clinics in Latin American. Overall error rates were similar between self- and travel-auditors; 94% of entries received the same assessment from self- and travel-auditors, and the majority (72%) of incorrect variables received the same corrections from both groups. Despite some discordance and mismatched corrections between self- and travel-auditors, our findings suggest that data audits carried out by local investigators can provide a viable alternative to travel-audits to investigate data quality.

Monitoring data quality in a multisite research network can be logistically challenging, costly, and time-consuming [10]. The time and costs have been well documented in the clinical trials space, where extensive source document verification is often required for government regulatory agency approval of new drugs and devices [15]. Cost-savings measures in clinical trials have prompted the rollout of “remote auditing,” where trials auditors log into the electronic medical records of distant hospitals to review and verify patient information [9,10]. Such solutions are not feasible in many global heath settings, however, where electronic systems are not designed for secure remote login capacity, large geographic distances produce high internet latency, patient charts are on paper, or internet is not available.

The CCASAnet cohort faces particular challenges because the sites are dispersed in seven countries throughout North and South America. Despite the demonstrated importance of data audits [1–7], constraints in both time and funding have limited the extent to which data audits have been performed in this network. The self-audits described in this manuscript allowed us to collect extensive data on all eight sites using fewer resources. The self-audit involved a two-hour online training session and compensation of $2000 US dollars to local investigators. Local sites were given two weeks to complete the self-audit, compared to the approximately two and a half days travel-auditors spent at each site. The number of entries audited was strikingly higher by self-auditors for lower cost and roughly equivalent resulting quality.

Although results were largely similar between self- and travel-auditors, the between-auditor discrepancies that we observed illustrate some challenges with determining correct values, even with an audit. Many audit decisions are not straightforward, and neither the self- nor travel-audits should be considered a gold standard. Self- and travel-auditors contributed insights into the reasons for mismatched corrections, and when shown the results, recommended establishing a better protocol for definitions of certain variables for data auditing and collection. Both sets of auditors felt there could be ambiguity in interpretations of specific variables (e.g., can CDC stage go from C to B, or once C is it always maintained as C?), which could contribute to mismatches. Audit entries were labeled as “correct” or “incorrect,” whereas there is often some ambiguity in the true value, for example, an auditor’s inability to find an original value in the source document does not necessarily imply that the original data were incorrect.

Our study has several limitations. Travel-auditors were unable to completely audit all records, and the subset of doubly audited records may not be fully representative of all records selected by the data coordinating center to be audited. This analysis excluded three patient fields of potential interest (clinical AIDS diagnosis prior to first visit (y/n), date of prior clinical AIDS diagnosis, and WHO stage) because they were incompletely audited, which poses limitations in extending these findings to these specific variables from patient records. At some sites, self-auditors may have been involved in the original data entry, which could potentially lead to under-reporting of errors. Sites were aware that their records could be externally audited, which we believe strengthened the quality of their self-audits and lessens concern of such under-reporting. In addition, sites were given a small amount of compensation for completing their self-audits. Self-audits without the possibility of external auditing or without compensation may perform differently. Only three sites were doubly audited, and sites that were not selected for a travel-audit may have had substantially different levels of concordance between self- and travel-audits.

Finally, our travel- and self-audit results may not be applicable to other settings. Settings with paperless data capture may require different source data verification techniques, such as detailed consideration of all text and data fields in an electronic health record, some of which may not have made it into the research database. We recognize that all our sites had prior experience with audits, were active research contributors, and engaged in data quality activities. If sites are unfamiliar with data quality concepts or when scientific misconduct or fraud is a potential concern, self-audits are unlikely to be an effective solution despite cost-savings.

Our study has several strengths. This study builds upon previous data quality initiatives in a diverse, multinational HIV cohort. Initial self- and travel-audit findings gauged the overall integrity of data at the three sites, while the comparison of doubly audited entries investigated the efficacy of the proposed self-audits to replace travel-audits in the future. The analysis built on a large dataset captured many facets of the patient record, allowing us to draw conclusions not just about the general quality of data but to also inspect the integrity of specific variables and forms. Additionally, antifraud precautions were incorporated into the self-audit methodology: (1) patient IDs were randomly selected by the CDCC-VU (not the sites) and (2) a random subset of the eight sites were chosen for a travel-audit, as well.

With similar overall error rates, findings suggest that self-audits are an effective approach for assessing data quality and should be considered in networks performing analyses using pooled HIV observational data. However, discrepancies observed between corrections illustrate challenges in determining correct values even with audits. For multisite collaborations, we recommend conducting baseline travel-audits. After the team is familiar with sites’ data quality and the audit process, we recommend regular audits, which may include self-auditing in a manner similar to that described here.

## Supplementary material

For supplementary material accompanying this paper visit http://dx.doi.org/10.1017/cts.2019.442. click here to view supplementary material
