# Supplementary material for: Self-audits as alternatives to travel-audits for improving data quality in the Caribbean, Central and South America network for HIV epidemiology
Source: J Clin Transl Sci. 2019 Dec 26;4(2):125–32. doi: 10.1017/cts.2019.442 (PMC7159809; doi:10.1017/cts.2019.442)
Supplement: Supplementary file 1 [file S2059866119004424sup001.docx]

**Supplementary Material**

Table S1. Data dictionary for CCASAnet variables.

| tblBAS | Contains basic patient information typically collected at enrollment. |
| --- | --- |
| record_id | Study ID |
| male_y  birth_d  hiv_diagnosis_d  mode  recart_y  aids_y  aids_d  aids_cl_y  aids_cl_d  baseline_d  pmtct  birth_mode | Sex  Birth date  HIV diagnosis date  Mode of transmission  Prior ART?  AIDS diagnosis prior to first visit?  Date of prior AIDS diagnosis  Clinical AIDS diagnosis prior to first visit?  Date of clinical AIDS diagnosis prior to first visit  First visit date at CCASAnet clinic  Received PMTCT as an infant? (pediatric sites only)  Birth mode (pediatric sites only) |
| tblLTFU | **Contains death and drop-out information collected during follow-up.** |
| drop_y | Has patient been dropped from cohort? |
| drop_d  drop_rs  death_y  death_d | Date of drop  Reason for dropping  Did the patient die?  Date of death |
| tblVIS | **Contains visit-related information.** |
| visit_d | Visit date |
| height  weight  cdcstage  whostage | Height (in cm)  Weight (in kg)  CDC stage  WHO stage |
| tblLAB_CD4 | **Contains CD4 lab measurement.** |
| cd4_d | CD4 date |
| cd4_v  cd4_per | CD4 value  CD4 percent |
| tblLAB_RNA | **Contains viral load lab measurement.** |
| rna_d | Viral load date |
| rna_v | Viral load value |
| tblART | **Contains antiretroviral therapy (ART) information.** |
| art_id | ART regimen |
| art_sd  art_ed  art_rs | Start date of ART regimen  End date of ART regimen  Reason for stopping ART regimen |
| tblCEP | **Contains clinical events including serious non-AIDS conditions.** |
| ce_d | Date of disease diagnosis |
| ce_id | Disease code |

The CCASAnet data structure roughly follows data exchange protocols outlined by the HIV Cohorts Data Exchange Protocol (HICDEP) and the International epidemiology Databases to Evaluate AIDS (IeDEA) (12-13).

Table S2. Comparing baseline and follow-up characteristics of patients who were only self-audited and who were self- and travel-audited

| **Variable** | **Self- and travel-audited (n = 65)** | **Self-audited only (n = 65)** | **P-Value** |
| --- | --- | --- | --- |
| **Age at first clinic visit** mean(sd) | 15.3 years (17.9) | 18.5 years (18.9) | 0.55 |
| **Age at death** mean(sd) | 18.0 years (21.2) | 31.3 years (24.5) | 0.36 |
| **Number of form entries** mean(sd) |  |  |  |
| ART regimen | 2.7 (2.5) | 3.6 (3.0) | 0.06 |
| CD4 labs | 17.4 (16.3) | 21.4 (19.2) | 0.09 |
| Clinical endpoints | 5.9 (9.2) | 6.6 (8.0) | 0.62 |
| Viral load labs | 17.8 (16.3) | 20.6 (18.1) | 0.20 |
| Visits | 43.4 (44.8) | 57.4 (58.1) | 0.15 |
| **Sex (% male)** | n = 41 (63%) | n = 37 (57%) | 0.52 |
| **Dead (% yes)** | n = 12 (18%) | n = 14 (22%) | 0.51 |
| **AIDS (% yes)** | n = 20 (31%) | n = 17 (26%) | 0.12 |
| **Prior ART (% yes)** | n = 10 (15%) | n = 9 (14%) | 0.83 |
| **Drop from cohort (% yes)** | n = 22 (34%) | n = 16 (25%) | 0.44 |
| **Reason for dropping*** |  |  |  |
| LTFU/ not known to be dead | n = 8 (36%) | n = 5 (31%) | Ref |
| Other | n = 8 (36%) | n = 7 (44%) | 0.77 |
| Transfer to another center | n = 6 (27%) | n = 4 (25%) | 0.77 |
| **Mode of transmission** |  |  |  |
| Heterosexual Contact | n = 7 (11%) | n = 13 (20%) | Ref |
| Homosexual/bisexual contact | n = 9 (14%) | n = 9 (14%) | 0.28 |
| Perinatal | n = 39 (60%) | n = 31 (48%) | 0.45 |
| Other | n = 2 (3%) | n = 2 (3%) | 0.86 |
| Unknown | n = 8 (12%) | n = 10 (15%) | 0.49 |

P-values are from the Wald tests for the coefficient effects in logistic regression models for whether the patient was included in the analysis, controlling for site. *proportions of reason for dropping are limited to subjects who were dropped.


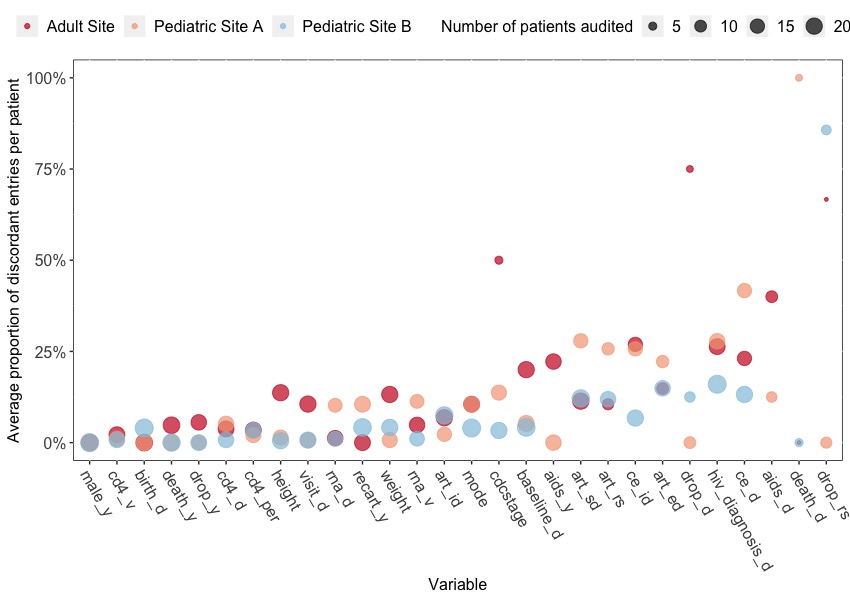


Figure S1. Average proportion of discordant entries per patient by variable by site (sized by number of audited patient records). For variables that are collected once, this is the proportion of patients whose entries were discordant. For variables that are collected more than once, this is the average of the per-patient proportion of entries that were discordant.
